# Calcifying Matrix Vesicles and Atherosclerosis

**DOI:** 10.1155/2017/7463590

**Published:** 2017-11-07

**Authors:** Dimitry A. Chistiakov, Veronika A. Myasoedova, Alexandra A. Melnichenko, Andrey V. Grechko, Alexander N. Orekhov

**Affiliations:** ^1^Department of Molecular Genetic Diagnostics and Cell Biology, Division of Laboratory Medicine, Institute of Pediatrics, Research Center for Children's Health, Moscow 119991, Russia; ^2^Laboratory of Angiopathology, Institute of General Pathology and Pathophysiology, Moscow 125315, Russia; ^3^Unit of Atherosclerosis Prevention, Centro Cardiologico Monzino, IRCCS, 20138 Milan, Italy; ^4^Federal Scientific Clinical Center for Resuscitation and Rehabilitation, 14-3 Solyanka Street, Moscow 109240, Russia; ^5^Institute for Atherosclerosis Research, Skolkovo Innovative Center, Moscow 121609, Russia

## Abstract

Artery calcification is a well-recognized predictor of late atherosclerotic complications. In the intima media, calcification starts with apoptosis of vascular smooth muscle cells (VSMCs) and the release of calcifying matrix vesicles with diameter of 0.5–15 *μ*m that can be observed microscopically. In complicated plaques, calcification is generally less frequent. Calcifying vesicles are released by proatherosclerotic VSMCs into the collagen-rich matrix. The vesicles can penetrate into the intima media and protrude into the arterial lumen and thereby may represent a potential cause of atherothrombosis. In calcified fibrolipid plaques, the rate of calcification is increased but is followed with healing of a lesion rupture and exhibited by further erosion and/or intimal thickening. Generally, calcification directly correlates with the apoptosis of VSMCs and macrophages accompanied by the release of osteogenic matrix vesicles. This is a hallmark of atherosclerosis-related apoptosis of VSMCs that is commonly released in plaque stabilization.

## 1. Introduction

So far, three major types of blood vessel calcification have been described, including proatherosclerotic arterial calcification, Mönckeberg calcification in the intima media, and infantile calcification [1]. Calcification of atherosclerotic lesions starts in the middle age and is prevalent in the elderly population. Clinical outcomes of the atherosclerosis involve unstable or stable angina, thrombosis, heart attack, stroke, and sudden coronary death. It has been found that lethal cases from coronary thrombosis were caused by acute coronary syndrome followed by erosion and formation of calcified nodules [2]. In this paper, we will focus on the mechanisms of arterial calcification associated with atherosclerotic disease.

## 2. Atherosclerotic Lesion Progression

Atherosclerosis progression is a dynamic process that spans through a substantial part of the lifetime and leads to the formation of unstable lesions followed frequently by acute thrombosis, which can be lethal or life-threatening. Atherosclerosis-associated thickening of the arterial wall starts with an increase in vascular smooth muscle cells (VSMCs) count and extracellular matrix (ECM) mass in the intima and media, paralleled by infiltration of inflammatory cells as a result of the adaptive immune response. Macroscopically visible plaques begin to develop as fatty streaks located in the subendothelial region of the arterial wall, which are infiltrated with lipid-laden macrophages [3]. However, the earliest events in the lesion development are associated with the formation of cell-free lipid pools located within the intima, but in the vicinity of the media, and characterized by the absence of VSMCs and enrichment in ECM matrix proteins [2].

At a more advanced stage, atherosclerotic plaque can be defined as fibroatheroma with a lipid-enriched necrotic core surrounded by a collagen-rich tissue. The early stage of fibroatheroma (early fibroatheroma) is characterized by infiltration of macrophages into the lipid core and focal loss of proteoglycans and collagen matrix [1]. Atherosclerotic plaque development has been described in detail in a number of early studies [4–6].

## 3. Microscopic Features of Atherosclerosis-Related Calcification

**Figure 1:**
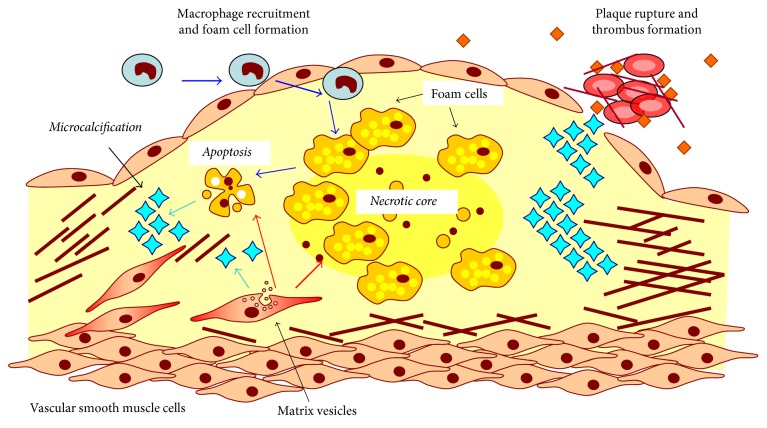
Pathways of microcalcification in the atherosclerotic plaque. The major sources of calcification are apoptotic cells derived from foam cells of macrophage or vascular smooth muscle cell (VSMC) origin and calcifying matrix vesicles released by VSMCs that lost their contractile phenotype and acquired increased synthetic and proliferative activity. Accumulating calcified plates can cause rupture of the plaque and thrombus formation.

Early stages of vascular calcification are initiated from the microcalcification (Figure 1). The calcifying particles released by VSMCs typically have a diameter less than 15 *μ*m [7]. Early proatherosclerotic calcification is marked by the presence of vesicles of less than 1 *μ*M in size. Arterial calcification usually occurs in the intima, close to the internal elastic lamina. The amount of intimal VSMCs are reduced in the basement membrane.

Although VSMC apoptosis is a major way of vascular calcification, microcalcification can also be caused by apoptotic death or the release of matrix vesicles by macrophages [8]. In macrophages, microcalcification could be detected by changes in cell morphology. This process can start in the necrotic lesion core, where macrophages are present as foam cells and contribute to the pool of dying and dead cells. In the necrotic core, calcification progresses and extends from the outer rim of the necrotic core into the collagen-rich matrix that envelops the core [1].

Atherogenic calcification leads to the formation of a calcified plaque that in turn forms calcified plates. The calcified plates can penetrate the tunica media into the lumen or grow in the opposite direction into the media [9]. When this occurs, this may lead to thrombosis, which contributes up to 14% of cases with carotid plaques [10].

Osteogenic formations are rare in the areas of arterial calcification that occur predominantly in heavily calcified vascular regions [1]. Vascular calcification shares several mechanisms with normal bone calcification and cartilage generation, including the expression of major proosteogenic factors, such as osteocalcin, osteoprotegerin, osteopontin, and bone morphogenic proteins- (BMP-) 2 and 4. However, a differential expression pattern of osteogenic proteins was observed in course of the plaque progression. For example, bone sialoprotein matrix and Gla protein (MGP) showed increased expression in the endothelial cells and VSMCs of the intima media of normal aorta [11]. Higher levels of osteonectin, osteopontin, and BMP-4 were detected in VSMCs. In advanced fibroatheromatous plaques, expression of osteoprotegerin, osteonectin, BMP-2, and BMP-4 was found in the shoulder regions. Expression of MGP, bone sialoprotein, osteopontin, osteocalcin, BMP-4, osteonectin, and osteopontin was reported in foam cells and in the lipid core. In fibrocalcific lesions that have collagen-enriched and calcified areas and in lesions with signs of chondrogenic and osteogenic formations, increased expression of osteonectin, osteopontin, BMP-2, and BMP-4 was demonstrated [11]. In addition, extensive microcalcification with increased expression of calcification markers, such as osteocalcin and BMP-2, was observed in patients with coronary artery disease.

Vascular calcification occurs in all atherosclerotic lesions independently of their arterial location. In peripheral arteries, atherogenesis can be found not only in the intima, but also in the media (so called Mönckeberg calcification) [12]. Compared to the carotid and coronary artery atherosclerosis, the peripheral disease exhibits signs of elevated collagen deposits and increased calcification [1]. In the Multi-Ethnic Study of Atherosclerosis (MESA), carotid artery calcification was shown to be present in 50% of participants with no clinical signs of atherosclerosis with the average age of 57.9 years [13]. A larger size of necrotic core with less calcification was reported in carotid atherosclerotic patients with stroke compared to the individuals who had ocular symptoms [14]. A total of 68% of subjects with asymptomatic carotid stenosis undergoing surgical carotid endarterectomy were found to have increased calcification compared with symptomatic patients (49%) [15].

## 4. Calcification and Lesion Vulnerability

Ultrasound examination showed that plaques with heavy deposits of calcium tend to be less vulnerable in changing the necrotic core size than lesions with less prominent calcification. However, in patients with stable coronary artery disease (CAD), spotted calcification was shown to correlate with increased atheroma size and progression [16]. In contrast with the atheroma volume, increased calcification does not enhance fibrous cap stress in stable or ruptured human coronary atherosclerotic plaques [17]. In CAD patients, study of autopsy samples showed a reverse correlation between the calcification rate and infiltration of coronary arteries by macrophages [18]. Furthermore, imaging studies conducted on patients with acute coronary syndrome (ACS) and autopsy studies of CAD patients indicated reduced calcium deposits in problematic lesions in comparison to stable plaques [19]. These observations suggest that calcification generally supports lesion stability. However, some studies showed that microcalcification could destabilize a plaque. For example, plaques with thin fibrous cap with sizes typically smaller than 10 *μ*m have increased chances of rupture [20]. Similarly, lesions with microcalcification and sizes exceeding 5 *μ*m were shown to be associated with increased plaque instability, whereas plaques with less microcalcification area (less than 5 *μ*m in diameter) were more stable [7].

Recently, a correlation between the coronary stenosis and calcification rate was shown. The calcification was significantly increased in individuals who died from heart attack with more than one coronary artery affected by atherosclerosis (stenosis of more than 50%) compared to those who died from noncardiac events. Meanwhile, coronary calcification is not associated with signs of plaque instability, which in turn suggests that calcification of coronary arteries could be helpful as a marker of ACS, but not of instable plaques [21].

However, age at onset of coronary atherosclerosis could serve as a predictor marker for calcification between patients with stable and vulnerable lesions. An inverse correlation was reported between age, lesion stability, and calcification [22].

## 5. Imaging Studies of Calcification

Computed-based tomographic angiography (CBTA) was suggested to be helpful for predicting further acute events in patients with intermediate cardiovascular risk [23]. Indeed, patients with heavy arterial calcium deposits exceeding 75 percentile have the risk of acute cardiovascular complications increased by 19-fold in comparison with those who have calcium score of less than 25th percentile [24]. Similarly, a follow-up study showed that patients had elevated low-density lipoprotein (LDL) cholesterol and no cardiovascular risk factors except for the presence of calcium in coronary arteries in comparison with individuals who have no arterial calcification [25]. In addition, absence of calcification in CAD patients was found to be associated with reduced risk of ACS [26].

In general, CBTA is able to properly detect calcification foci that exceed 1.03 mm^2^ in diameter [27]. Microcalcification areas of smaller size could not be detected by computed tomography in early lesions [28]. CBTA could only identify calcified areas when calcium deposition occurred in fiber-rich atheromas in the frontier between the tunica intima and tunica media [29].

## 6. Impact of Race and Gender Differences on Arterial Calcification

Several studies suggest the existence of gender differences in cardiovascular disease development. The onset of CAD is delayed in females as compared to males by 10–15 years, indicative of a putative protective action of estrogen [30]. Administration of estrogen in women (aged 50–59) with CAD in a 7-year follow-up study was shown to be protective against calcification compared with a placebo group (Manson et al. 2007). In postmenopausal women who died from CAD, the calcification rate was 3-fold higher than in premenopausal women [18], again suggesting protective activity of estrogen.

Race differences can also contribute to the degree of calcification. In the MESA study, asymptomatic participants of 45–84 years of age had the relative risk of calcification estimated as 0.78 in blacks, 0.85 in Hispanics, and 0.92 in Asians as compared to the participants of Caucasian heritage [31]. Similarly, the analysis of autopsy samples from white Caucasians who died from CAD showed that the degree of calcification was higher in comparison to patients of black heritage for all decades [29]. This observation was also confirmed by tomography investigations [32]. It is possible that race-related differences in calcification are defined by multiple factors. Bone mineral density, which is inversely correlated with arterial calcification, is higher in individuals of black heritage in comparison to Caucasians [33]. Genetic factors could also contribute to racial differences in calcification. For example, GRB2-associated-binding protein 2 (GAB2), a signaling transducer, which is involved in the interaction between receptor tyrosine kinases (RTKs) and non-RTK receptors, was found to be less expressed in blacks and in subjects with reduced calcification [34].

## 7. Calcification, Vascular Remodeling, and Arterial Stenosis

Arterial calcification was shown to strongly correlate with lesion formation and expansion but demonstrated a weak association with arterial stenosis [35]. Since calcification could serve as a good predictor of further cardiovascular complications, it is not surprising that a strong correlation between calcification and lesion size can be observed. Glagov et al. [36] showed that lumen area does not reduce when a plaque extends over 40% of the internal elastic lamina area. In arterial stenosis, a compensatory mechanism called vascular remodeling takes place. A compensatory enlargement of arterial wall could account for the main reason of the poor correlation between calcification and the lumen area. Another explanation could come from the lesion morphology, since fibrotic plaques are inversely associated with arterial remodeling [29]. Arterial stenosis can take place in advanced plaques when compensatory mechanism of vascular remodeling becomes insufficient.

A significant correlation was also observed between the percentage of stenosis and the degree of calcification [29]. Cured lesion ruptures are often detected in advanced stenotic areas and are associated with increased calcification [28].

## 8. Vascular Calcification Mechanisms

As mentioned above, atherosclerotic intimal calcification is different from medial Mönckeberg calcification. Inflammation and lipid deposits do not contribute to calcification. On the other hand, diabetes mellitus and end-stage renal disease were shown to be associated with medial calcification [12]. Mönckeberg calcification first occurs in the media and in the internal elastic lamina followed by medial VSMC. The calcification is accompanied with loss of the contractile phenotype by VSMCs associated with downregulation of expression of smooth muscle markers, such as *α*-actin and SM-22*α*, and induction of expression of calcifying markers, such as osteopontin, Runt-related transcription factor 2 (RUNX2), osteocalcin, and alkaline phosphatase. MGP-deficient mice showed spontaneous arterial medial calcification, suggesting the involvement of MGP in medial calcification [37].

Medial and intimal calcifications have differentiated molecular mechanisms that are explained by several hypotheses. According to one of them, the main mechanism of intimal calcification is the depletion of anticalcifying agents, including fetuin, osteopontin, and pyrophosphates in the vascular tissue. Induction of expression of bone proteins, such as BMP-2, osteopontin, and osteocalcin, in the vascular wall and the presence of ossification and cartilage in vessel walls suggest ectopic mineralization [38]. Another explanation is that VSMCs may acquire an osteogenic phenotype with VSMC mineralization that occurs in a manner similar to bone formation. VSMCs release extracellular vesicles (or matrix vesicles), which became calcified when deposition of hydroxyapatite crystals occurs within the matrix vesicles [39]. This is followed by the induction of VSMCs apoptosis in calcified extracellular microenvironment and calcification of macrophages [40]. Both of these concepts provide explanation for the development of intimal mineralization. Apoptosis of VSMCs and macrophages was shown to be involved in proatherosclerotic intimal calcification [41, 42] and was shown to increase calcification* in vitro* [43].

Apoptosis is highly prevalent in the lipid core in type IV plaques and equally distributed between the fibrous cup and lipid core in type V plaques. In ruptured lesions, apoptotic zones could be equally observed in the fibrous cap, lipid core, and area underlining the rupture [44]. In fatty streak lesions, VSMC apoptosis is a rare event [41].

In advanced lesions, the necrotic core is almost devoid of cells as a result of cell death or plaque hemorrhages that could precede the expansion of the necrotic core [45]. The area that surrounds the necrotic core is highly infiltrated by macrophages with signs of apoptosis [46]. Compared with other lesion areas, the necrotic core is enriched with apoptotic bodies [47].

In cultured human VSMCs, induction of calcification was observed in apoptotic sites [42]. Apoptosis preceded calcification of VSMCs, and its inhibition could prevent calcification [42]. In apolipoprotein E- (apoE-) deficient mice with apoptosis of VSMCs induced by SM22*α*-human diphtheria toxin receptor, Clarke et al. [40] showed that low level of apoptosis increases calcification in early and late unstable lesions. Indeed, apoptotic bodies released by proapoptotic VSMCs play a prominent role in the induction of vascular calcification.

Hyperphosphatemia could also serve as inducer of calcification. Bovine aortic valve interstitial cells treated with high concentrations of inorganic phosphate (3 mM) showed signs of calcification and induction of alkaline phosphatase [48]. Given that the levels of calcium and phosphate are precisely controlled in biological fluids, deregulation of this balance could lead to ectopic calcification.

There remain many unanswered questions regarding the origin, mechanisms, and physiological significance of atherosclerotic calcification. However, a marked progress was done in studying mechanisms of bone formation. Improved understanding of this process strongly supports the concept that suggests the similarity between the mechanisms of bone formation and vascular mineralization. In apoE-deficient mice, reprogramming of VSMCs towards osteochondrogenic or chondrocytic differentiation and their involvement in atherosclerotic calcification was observed [49]. However, true ossification characterized by the presence of osteoblasts and osteoclasts is very uncommon in blood vessels. Also, formation of cartilage tissue in the arterial wall was rarely observed [50].

The location of calcification plays an important role in plaque progression. In advanced plaques, even small calcifications of the thin fibrous cap may lead to plaque destabilization and rupture. By contrast, large calcification areas in the intimal-medial boundary do not contribute to lesion instability but may be involved in the development of stable atherosclerotic disease.

## 9. Conclusion

Only a weak correlation between arterial calcification and arterial stenosis could be observed. Intimal thickening starts with the increase of VSMC count as well as proteoglycan and collagenous matrix without the inflammatory cells infiltration. This type of thickening is called adaptive intimal thickening (AIT) and is caused by changes in blood flow. The earliest progressive lesion is pathological intimal thickening (PIT) characterized by appearance of cell-free lipid intimal pools close to the media where VSMCs are absent but proteoglycans and lipid deposits are abundant.

Coronary calcification occurs randomly in AIT/fibrous plaques but almost always could be met in PIT lesions. In PIT, mineralization could be seen as microcalcification ranging from 0.5 to 15 *μ*m in size. In early fibroatheromas, when macrophages enter the necrotic core and become apoptotic, the size of calcified areas exceeds 15 *μ*m. The assemblage of calcified areas commonly happens in fibroatheromas and sporadically in PIT/fibrous lesions. During plaque progression, calcified areas grow and generate calcified sheets, a marker of stable and fibrocalcific lesions. Compared to unstable plaques, stable lesions are more heavily calcified.

The mechanisms of vascular calcification should be better understood with using animal models and new molecular and image techniques. In the future, strategies to control or remove calcification may enable us to reduce the burden of atherosclerotic disease.
